# Advanced Data Processing of Pancreatic Cancer Data Integrating Ontologies and Machine Learning Techniques to Create Holistic Health Records

**DOI:** 10.3390/s24061739

**Published:** 2024-03-07

**Authors:** George Manias, Ainhoa Azqueta-Alzúaz, Athanasios Dalianis, Jacob Griffiths, Maritini Kalogerini, Konstantina Kostopoulou, Eleftheria Kouremenou, Pavlos Kranas, Sofoklis Kyriazakos, Danae Lekka, Fabio Melillo, Marta Patiño-Martinez, Oscar Garcia-Perales, Aristodemos Pnevmatikakis, Salvador Garcia Torrens, Usman Wajid, Dimosthenis Kyriazis

**Affiliations:** 1 Department of Digital Systems, University of Piraeus, 18534 Piraeus, Greecedimos@unipi.gr (D.K.); 2 Facultad de Informática, Universidad Politécnica de Madrid, 28040 Madrid, Spain; aazqueta@fi.upm.es (A.A.-A.); mpatino@fi.upm.es (M.P.-M.); 3 Athens Technology Center S.A., 15233 Athens, Greece; t.dalianis@atc.gr (A.D.); m.kalogerini@atc.gr (M.K.); 4 Information Catalyst, S.L., 46800 Xàtiva, Spain; jake.griffiths@informationcatalyst.com (J.G.); oscar.garcia@informationcatalyst.com (O.G.-P.); usman.wajid@informationcatalyst.com (U.W.); 5 Innovation Sprint, 1200 Brussels, Belgium; kkostopoulou@innovationsprint.eu (K.K.); skyriazakos@innovationsprint.eu (S.K.); dlekka@innovationsprint.eu (D.L.); apnevmatikakis@innovationsprint.eu (A.P.); 6 LeanXscale, 28223 Madrid, Spain; pavlos@leanxcale.com; 7 Engineering Ingegneria Informatica SpA, 00144 Rome, Italy; fabio.melillo@eng.it; 8 Hospital de Denia Marina Salud S.A., 03700 Alicante, Spain; garcia_saltor@gva.es

**Keywords:** machine learning, ontologies, semantic web, holistic health records, data science, primary and secondary data, pancreatic cancer, wearables

## Abstract

The modern healthcare landscape is overwhelmed by data derived from heterogeneous IoT data sources and Electronic Health Record (EHR) systems. Based on the advancements in data science and Machine Learning (ML), an improved ability to integrate and process the so-called primary and secondary data fosters the provision of real-time and personalized decisions. In that direction, an innovative mechanism for processing and integrating health-related data is introduced in this article. It describes the details of the mechanism and its internal subcomponents and workflows, together with the results from its utilization, validation, and evaluation in a real-world scenario. It also highlights the potential derived from the integration of primary and secondary data into Holistic Health Records (HHRs) and from the utilization of advanced ML-based and Semantic Web techniques to improve the quality, reliability, and interoperability of the examined data. The viability of this approach is evaluated through heterogeneous healthcare datasets pertaining to personalized risk identification and monitoring related to pancreatic cancer. The key outcomes and innovations of this mechanism are the introduction of the HHRs, which facilitate the capturing of all health determinants in a harmonized way, and a holistic data ingestion mechanism for advanced data processing and analysis.

## 1. Introduction

During the last decade, the development and utilization of cutting-edge technologies, such as IoT, ML, and Artificial Intelligence (AI), have experienced exponential growth in different domains [1,2,3]. The insights of a recent survey indicate that most of the emerging technologies and trends are three to eight years away from reaching widespread adoption but are the ones that will have significant impact during the next years [4]. Although many of these technologies are still in their infancy, organizations and businesses that adopt and embrace them early will be able to gain significant advantages against their competitors. Some of these technologies, such as ML, Deep Learning, Edge AI, Human-Centered AI, Synthetic Data, and Intelligent Applications, can significantly impact the healthcare sector, among other domains. In that direction, remarkable outcomes and results have been recently achieved through the implementation and utilization of advanced and sophisticated ML and AI algorithms and applications in various tasks within the healthcare domain. Noteworthy achievements in the tasks of personalized diagnostics [5], disease early risk identification [6], and personalized medicine [7] have been realized by employing ML models aiming to introduce enhanced and personalized prevention and intervention measures. However, the processing and analysis of vast numbers of datasets, ranging from medical images to secondary data collected from wearables and sensors, and from genetics to genomics, have revealed the need for the utilization of more complex algorithms when aiming to identify hidden patterns and integrate heterogeneous data in an optimum way. Hence, applications that are based on the concepts of Deep Learning and Artificial Neural Networks (ANNs) have gained wide adoption [8]. Leveraging the power of Deep Learning for automatic and unsupervised discovery of representations, these applications introduced more efficient and accurate solutions targeting the extraction of actionable insights, especially from large-scale datasets that have not been extensively curated [9]. This success spans across diverse tasks within the healthcare domain, including medical image classification [10], segmentation of Magnetic Resonance Imaging (MRI) data [11], semantic interoperability through applications of Natural Language Processing (NLP) [12], and hospital readmission predictions [13], where different types of ANNs, such as Convolutional Neural Networks (CNNs) [14] and Recurrent Neural Networks (RNNs) [15], have realized exceptional results contrary to ML-based alternatives.

The integration and utilization of these technologies can enhance the provisioning of remote diagnostics, as well as of early diagnosis and pre-diagnosis of critical diseases [16]. The high demand for remote patient monitoring and personalized healthcare has vastly improved the health analytics techniques and their implementation in healthcare systems. Emphasis on health analytics is also supported by the increasing utilization of wearables and the Internet of Medical Things (IoMT), which provide easy access to a large pool of health-related data. It should be noted that wearable devices are projected to grow at a 9.1% CAGR and IoMT at 23.70% between 2023 and 2032 [17]. The latter highlights the emerging need for the adoption of integrated Deep Learning and Edge AI techniques and approaches as, in the modern healthcare domain, the collection, processing, and analysis of the data will be performed more frequently on local devices rather than relying entirely on centralized cloud servers. In that context, Deep Learning techniques contribute significantly to the processing and interpretation of divergent and integrated data at the edge. However, their complexity and increasing need for sustainable cost- and time-effective solutions have led to the introduction of a range of techniques aimed at reducing network complexity and improving the efficient integration of Deep Learning models in edge devices [18]. Different techniques such as the majority voting [19] and the non-local adaptive hysteresis despeckling (NLAHD) techniques [20] have been recently introduced. Coupled with the utilization of Deep Learning models in healthcare-related tasks such as the early detection of acute lymphoblastic leukemia (ALL) [19] and the analysis and noise reduction of ultrasound images [20], they managed to introduce faster, efficient, and comprehensive applications. Sensors, wearables, and IoMT devices can be empowered by the integration between Deep Learning and Edge AI techniques, fostering the identification of intricate patterns, hidden anomalies, and complex representations. The latter leads to more efficient and autonomous decisions in real time without the need for constant connectivity to the cloud and centralized centers [21].

However, the healthcare domain faces various challenges related to the diversity and variety of data, the huge volume of data, and the distribution of data; thus, there is an ever-increasing demand from healthcare organizations for the implementation and utilization of new solutions and data-centric applications that can help them gain actionable insights from their data [22]. Data have long been a critical asset for medical organizations, hospitals, governments, and other stakeholders in the healthcare domain. The massive investments by the healthcare industry into new technologies and the rapid growth in the usage of cloud computing, mobile computing, medical devices, IoMT, and AI are some of the key factors that promote the need for enhanced and state-of-the-art health data-processing solutions [23]. In this respect, health-related data-processing solutions increasingly focus on exploiting value from primary data (coming from established data sources such as lab results, genomics, and family history) or secondary data (coming from IoMT devices that automatically measure and monitor in real time various medical parameters in the human body). The integration of primary and secondary data has revealed the potential for greater insights for healthcare and health-related decision making [24]. Even if, for collecting prospective and retrospective clinical data, there already exists a plethora of methods and techniques for automatically capturing such data in batches [25], this is not the case for the ingestion of streaming data, which has come to the attention of research and development during the last five (5) years [26,27]. As a result, current healthcare and assisted living solutions need to be enhanced to support the processing of primary and secondary data since citizens have increasing access to personal IoMT devices that can monitor individual parameters (e.g., heart rate, sleeping condition) and track their daily activities (e.g., distance walked, calories burned).

However, the existing hospital systems, EHRs, and IoMT devices most of the time are surrounded by high levels of heterogeneity since they have diverse formats, capabilities, functionalities, and characteristics. Hence, to effectively work with both the primary and secondary data, there are still challenges with regard to the standardization, qualification, and interoperability of the different types of data that are used by the existing healthcare systems. In that direction, there is even a growing demand for the development of methodologies and procedures for the standardized integration, processing, and analysis of heterogeneous data derived from divergent data sources and devices in modern Healthcare Information Systems (HISs). Such improvements can lead to enhanced diagnostics and care strategies, as well as to the extraction and utilization of actionable value and knowledge from available data in the healthcare domain.

What is more, timely diagnosis is very important when it comes to critical diseases, such as cancer, and especially to pancreatic cancer, which is uncurable and usually lacks clear symptoms at its early stages [28]. Understanding the underlying causes or risk factors can help to identify individuals at high risk of developing pancreatic cancer. From there, specific measures (preventions and interventions) can be introduced to reduce the risks, e.g., those that work on modifiable risk factors that relate to lifestyle, behaviors, and social interactions (e.g., reduction in smoking, alcohol, obesity, red meat consumption and increasing intake of vegetables, fruit, and regular physical exercise) [29]. Early identification of the modifiable risk factors of pancreatic cancer relies on healthcare professionals (HCPs) possessing sufficient knowledge, age-appropriate care programs, and community-based approaches aiming to provide specialized, multidisciplinary services both in terms of prevention of and interventions for diverse cancer-related factors. However, a significant gap still remains in the delivery of stratified healthcare because current approaches often take a one-size-fits-all approach [30]. Personalization implies a level of precision that seeks to treat the patient as opposed to the disease, taking into account, as an example, comorbidities, genetic predisposition, and environmental factors. The lack of integrated data (e.g., lifestyle data, Patient-Reported Outcome Measures (PROMs), Patient-Reported Experience Measures (PREMs), and genomic data) from patients that would allow clinicians to make personalized decisions as part of their clinical decisions limits the effectiveness of prevention strategies. Lack of integrated health data also hampers the potential of patient-centric interactions between HCPs, healthcare authorities, patients, and caregivers, as well as the potentials of advanced technologies, such as AI, for accurate risk prediction, prevention, and intervention [31].

Considering all these challenges, by effectively gathering, standardizing, and analyzing both primary and secondary data, collective community knowledge and personalized health insights could be extracted. The latter is facilitated by the collection, integration, and analysis of information from different sources concerning individuals for the provision of actionable insights at the point of care. To address gaps and requirements in individualized or personalized healthcare, this article introduces a data-processing mechanism that aims to integrate heterogeneous data sources to realize Holistic Health Records (HHRs) that can provide complete, integrated data views. To effectively construct the HHRs, the platform develops various data management techniques by integrating Semantic Web and ML techniques covering the complete data lifecycle, from the collection of the heterogeneous data to their aggregation, processing, and harmonization.

The mechanism introduced in this article has been evaluated based on a real-world scenario that provides different datasets, ranging from hospital-retrieved data to data from wearables, questionnaires, and mobile applications, proving its wider applicability and overall efficiency. The mechanism is developed in the context of the EU-funded project iHelp, which seeks to deliver a novel, personalized healthcare framework and digital platform that can enable the collection, integration, and management of primary and secondary health-related data [32]. Leveraging the knowledge and insights derived from these integrated data, the platform further provides advanced AI-based models, decision support, and monitoring systems to help with early identification and mitigation of pancreatic-cancer-related risks.

Thus, the goal of this paper is to evaluate the implementation of an advanced data-processing and harmonization mechanism with a specific focus on the real world that leverages data related to pancreatic cancer. Hence, this paper includes contributions such as:

- The introduction of an end-to-end and holistic reference architecture and data ingestion mechanism for advanced data processing and analysis in a modern HIS;
- A set of practical recommendations and implementations for the integration of techniques from the domains of data science, ML, and the Semantic Web;
- The realization of the HHR data model through the integration, standardization, and harmonization of primary and secondary data;
- Analysis and discussion of the industry-centric challenges and problems that researchers in the healthcare domain face with regard to data processing and analysis, such as data being available in divergent formats and semantic non-interoperable data.

The remainder of the paper is structured as follows. Section 2 describes the overall architecture of the proposed mechanism, depicting all of its incorporated components and the integration approach applied among them to achieve improved healthcare data integration and analysis. Section 3 evaluates the reference implementation of the mechanism against a real-world healthcare scenario, whereas Section 4 discusses the effectiveness of the current research work and its overall contribution as well as outlines any future work. Finally, Section 5 concludes this article.

## 2. Materials and Methods

**Figure 1:**
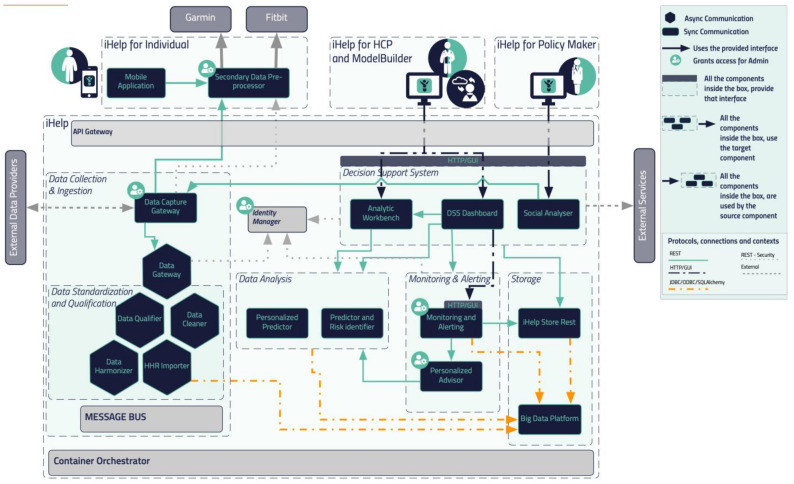
Overall architecture.

The flowchart and reference architecture of the overall iHelp platform are depicted in Figure 1. More specifically, the platform consists of five (5) different building blocks or sub-mechanisms: (i) Data Collection and Ingestion, (ii) Data Standardization and Qualification, (iii) Data Analysis, (iv) Monitoring and Alerting, and (v) Decision Support System. The integration of these different building blocks results in end-to-end integration and exploitation of the raw data through this novel and holistic platform [32]. In the context of this research work, we examine and evaluate the application of the first two (2) building blocks, i.e., Data Collection and Ingestion and Data Standardization and Qualification. It should be noted that the secondary or streaming data referred to in this article correspond to the data collected from Garmin wearable devices, whereas the primary or batch data correspond to the historical personal data of the individuals as provided by the Hospital de Dénia—Marina Salud (HDM), in line with the respective approval and decision of the Ethical Committee.

### 2.1. Reference Architecture

**Figure 2:**
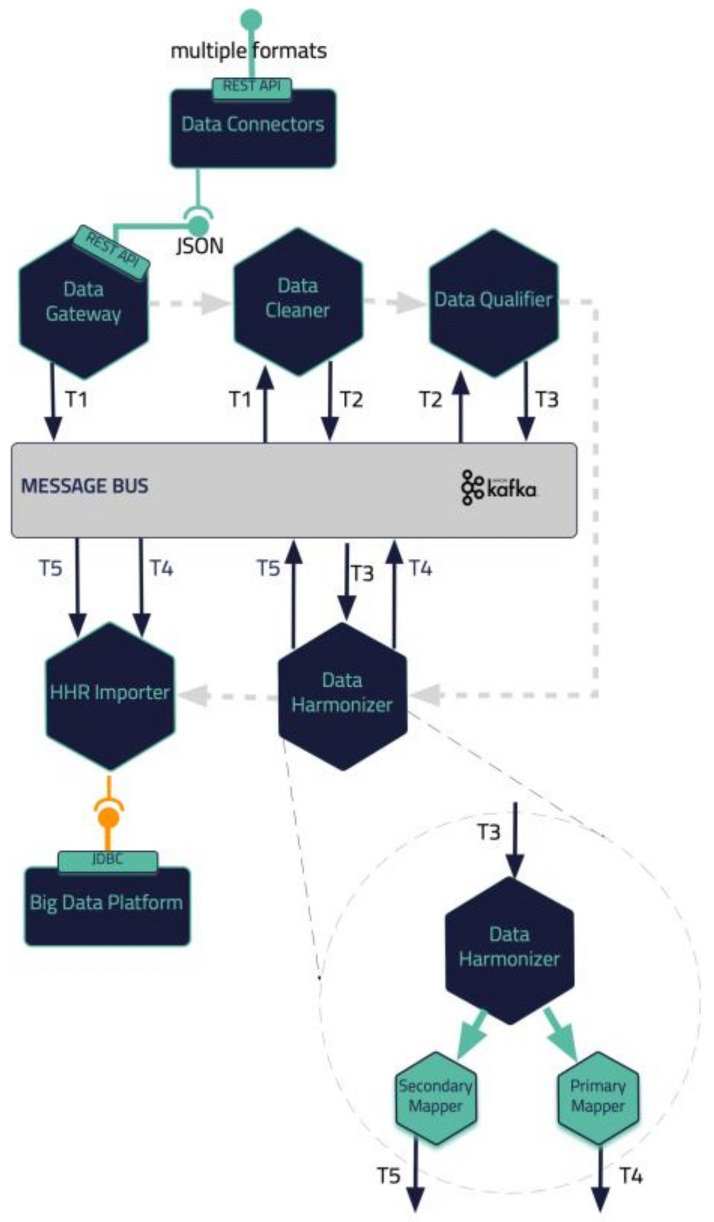
iHelp Data Ingestion Pipeline.

In this section, a blueprint of the proposed iHelp platform, developed in the context of the iHelp project [32], is presented, along with the internal process that takes place for its seamless interaction and integration with either secondary data sources (i.e., wearable devices) or primary data sources (i.e., hospital systems and databases), as depicted in Figure 1. As described previously, five (5) different building blocks and phases are incorporated in the iHelp platform [32]. It is characterized as a reference architecture since it is presented in a high-level, abstract, logical form, which provides a blueprint for the implementation of different functionalities such as AI-based healthcare analytics. In more detail, the platform initially consists of the sub-mechanisms of Data Collection and Ingestion, through which it may connect to heterogeneous data sources and gather their data, and Data Standardization and Qualification, which can process and harmonize the external healthcare data it receives and store them in its internal datastore. These two building blocks represent the end-to-end Data Ingestion Pipeline of the iHelp platform, as depicted in Figure 2. The software components incorporated in this pipeline are the Data Capture Gateway, Data Cleaner, Data Qualifier, Data Harmonizer, and HHR Importer, which consume data from one and produce them for the other by utilizing the capabilities of the Kafka message bus, which is further described in the next sub-section.

### 2.2. Integration Approach

In the integration of all these different components, the open-source Kafka and Kubernetes tools are utilized, providing a containerized approach for integrating the iHelp platform. The latter enables the deployment of this platform in different environments and infrastructures, showcasing its interoperability and improved adaptability in any deployment environment, e.g., in stakeholders’ servers and premises. The manifests that are developed as part of the deployment scripts contain all the needed components and respective installation prerequisites to establish and deploy the platform as a whole. On top of this, it should be noted that the iHelp has already been deployed and evaluated for its functionality and performance in the premises of two different hospitals (in the EU) in the context of the iHelp project [32]. 

To facilitate seamless and reliable data exchange between different components such as the two first sub-mechanisms (i.e., Data Collection and Integration and Data Standardization and Qualification), the iHelp platform uses Apache Kafka [33]. Kafka is a message broker and stream processor that allows the publication, subscription, archiving, and processing of streams of data/records in real time. It is specially designed to manage data streams from multiple sources by distributing them to multiple consumers. In this way, Kafka facilitates the collection and processing of both primary and secondary data that are ingested into the introduced mechanism. 

Apart from the use of Kafka as the platform’s message broker mechanism, the Kubernetes platform is utilized [34] to provide DevOps services. Kubernetes (K8s) is an open-source platform that automates Linux container operations. The integration between K8s and Kafka results in the simplification of the deployment of Kafka brokers as containerized pods as each Kafka broker can run as a separate pod, ensuring the scalability, fault tolerance, and availability of the overall approach. On top of this, microservices can be deployed to easily consume and produce data for Kafka topics, allowing for real-time data processing and analysis of the processed data in the context of the project. Finally, K8s eliminates many of the manual processes involved in deploying and scaling containerized applications and allows management of host clusters, which run containers easily and efficiently, and, for enhanced management of the K8s cluster, the Rancher tool is utilized.

### 2.3. Data Collection and Ingestion Pipeline

Health data can result from clinical tests performed invasively on samples taken from the patients’ bodies, or non-invasively using modern depicting techniques. Such data, obtained in a clinical setting, are of paramount importance and are termed as primary but certainly do not form the complete spectrum of health data [35]. Today, the importance of environmental factors, diet, and living habits is well established. The patients’ living habits can be enumerated using data attributes about their lifestyle, obtained in their natural environment, outside the clinical setting. These types of data are termed secondary since they correspond to health but are not determinists of typical health systems.

The Data Collection and Ingestion building block in the iHelp platform is responsible for the integration, anonymization, and verification of the primary and secondary data. Depending on the data source type that is connected and the corresponding method that must be used for ingesting its data (i.e., streaming collection for unknown sources and batch collection for known sources), this (i.e., Data Collection and Ingestion) sub-mechanism utilizes different connectors of the Data Capture Gateway as its main interfacing component.

**Figure 3:**
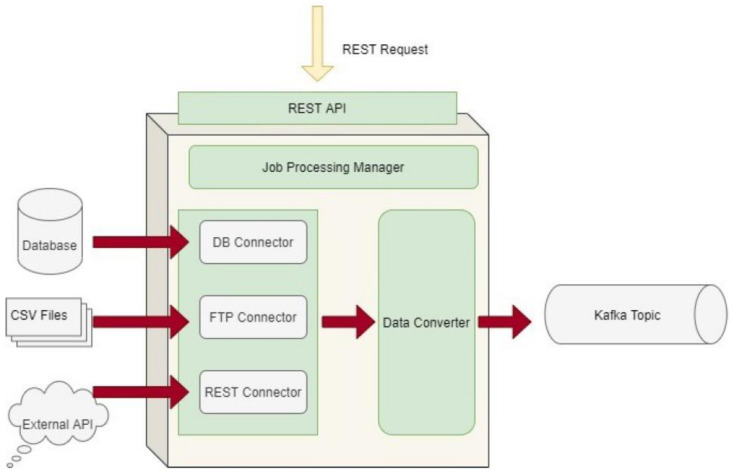
Data Capture Gateway overview.

The Data Capture Gateway is the component that can be considered as the interface between the iHelp integrated platform and the external data sources, both primary and secondary, from which it captures the data to be pushed into the established Data Ingestion Pipeline. The Gateway implements a standalone Java process, or a microservice, that takes care of connecting to the various external data sources and sends the data to an intermediate Kafka topic so that the data can be retrievable from the other functions in the Data Ingestion Pipeline. As such, it also provides REST APIs, which are used to initiate data capture activities or schedule them for a later or a periodic execution. The REST APIs of the Gateway are deployed into a servlet container; however, they make use of the core functionalities of the Data Ingestion Gateway, and, therefore, both the REST APIs and the code implementation are inside the single Java process. Regarding the schema of the datasets, this is translated into an Avro Schema compatible format by the Data Converter sub-function in order to boost the interoperability and has a well-known standard to be further used by other functions involved in the data ingestion process. A high-level overview of the different software elements of this initial design is depicted in Figure 3.

As the Data Capture Gateway captures data from the supported primary data sources, it forwards them into a common Kafka topic, from which it can be used by different components in the data pipeline. As has been described in the previous sub-sections, all software components that are involved in the Data Ingestion Pipeline are interchanging data through Kafka broker.

With regard to the secondary data, they comprise attributes that enumerate different important aspects of the way the patients live their lives. The attributes are grouped in the physiological, psychological, social, and environmental categories [36].

The physiological attributes are concerned with the human body, its activities, and adverse events, e.g., steps walked, distance walked, elevation (or floors climbed), energy dissipation, and time spent in different activity intensity zones and performing exercise activities (walking, running, cycling, etc.), as well as their distribution in the day. They are mostly measured using activity trackers. Attributes related to the functioning of the heart include the continuous measurements of the heart rate variability and the time spent in different heart rate zones, as well as the daily resting heart rate measurement. Sleep-related attributes include continuous measurements of the time spent in the different sleep stages (awake in bed, light, REM, deep sleep). Other physiological attributes like symptoms of interest, weight, and nutrition can be self-reported by the participant using widgets on a mobile app or questionnaires.

The psychological attributes refer to the emotions of the patients. They are mostly reported (although audiovisual or text-based emotion detection is possible) and include emotional state self-assessment using questionnaires or standardized reports from professional therapists.

The social attributes can be measured indirectly based on the usage of the mobile phone (diversity, duration, frequency of calls) and social media (diversity, number, frequency of interactions). More direct information can be reported using questionnaires on activities with others or can be obtained in conversation with a digital virtual coach or mobile app.

The environmental attributes include reported environmental indicators for the assessment of the quality of life. Measurements of living environment quality can be obtained by integrating relevant commercial devices (e.g., for air quality analysis), or by integrating with data services that report the Air Quality Index or weather details at the patients’ locations.

**Figure 4:**
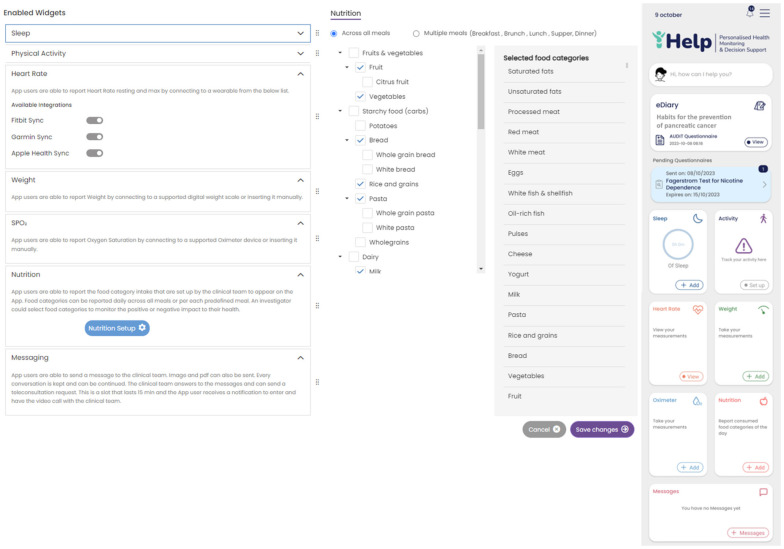
Setting up an iHelp study in Healthentia. Widgets are selected (**left**), and the nutrition widget is customized to include the food categories of interest (**middle**), resulting in the main dashboard of the mobile app (**right**).

Secondary data collection can be performed by the patients in their own everyday setting using a mobile application. The Healthentia mobile application, developed by Innovation Sprint, Brussels, Belgium, was selected to be utilized in the context of this research work. This mobile application offers interoperability between different mobile and wearable devices and allows the capture of data concerning all the abovementioned health determinants and categories [37]. Regarding the use of this application, at first, the corresponding portal is used to define the mobile app functionalities and the settings applied for a particular clinical study. This step results in the setup of the main application dashboard, as depicted in Figure 4. The data that are captured for each specific individual of the study are then transported in the iHelp platform through the secondary data connector of the Data Capture Gateway. From the Gateway, the data are forwarded to the internal Data Ingestion Pipeline for their further processing, cleaning, and transformation to the corresponding HHR data model. 

### 2.4. Data Modeling and Specification of Holistic Health Records

For addressing interoperability challenges, it is of paramount importance to develop adaptable and standardized data structures, which are termed as Holistic Health Records (HHRs). The HHR model is developed using existing models as a guide, with specific focus on the HL7 FHIR standard [38]. Although the HL7 FHIR standard is still in development and primarily designed to represent clinical data, it incorporates the capability to represent a broader range of data going beyond clinical information, e.g., streaming data originating from sensors. In this respect, the HHR model is engineered to be versatile and adaptable to various contexts thanks to the flexibility offered in the HL7 FHIR standard.

**Figure 5:**
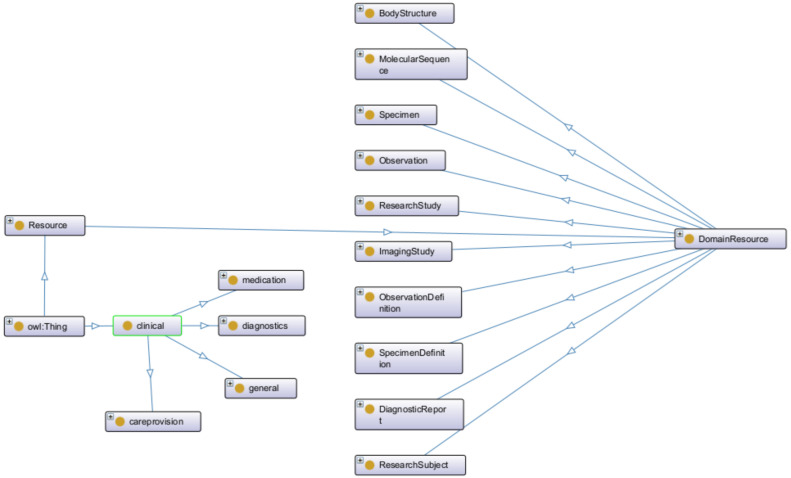
FHIR-based ontology sample.

Regarding the construction of the HHR model, the data gathered from the hospitals were initially grouped into medical categories for easier analysis of the concepts such as Pathology, Medication, etc. Then, every concept was mapped to the most relevant FHIR entity resources. The FHIR entity resources mostly used were Person, Observation, Condition, Procedure, Encounter, MedicationAdministration, etc. Any concept not directly mapped to an FHIR element resource was modeled by exploiting the standard mechanism provided by the FHIR standard, the Extensions, as an FHIR Extension inside the most relevant FHIR element mentioned, thus creating a separate Profile for these elements. Similarly, the non-standardized values of the hospital’s data attributes were translated, following the HCP’s knowledge, into standard SNOMED concepts [39]. If an attribute did not have a direct representation in SNOMED, it was included in the iHelp FHIR CodeSystem as a custom element. The representation of the iHelp conceptual model was achieved by using the TTL ontology syntax format, utilizing the FHIR ontology and the official guidelines in relation to creating FHIR CodeSystems, Extensions, etc. An instance of this ontology is depicted in Figure 5; as it is exported from the Protege tool, it showcases the relationships of the Clinical and Resource OWL classes. For example, the Clinical class is used to group clinical resources and has several subclasses like Medication, Diagnostics, General, Careprovision. In addition, the Resource class represents the base resource type and has one main subclass (DomainResource), through which the main health classes like Specimen, Observation, etc., are defined. 

**Figure 6:**
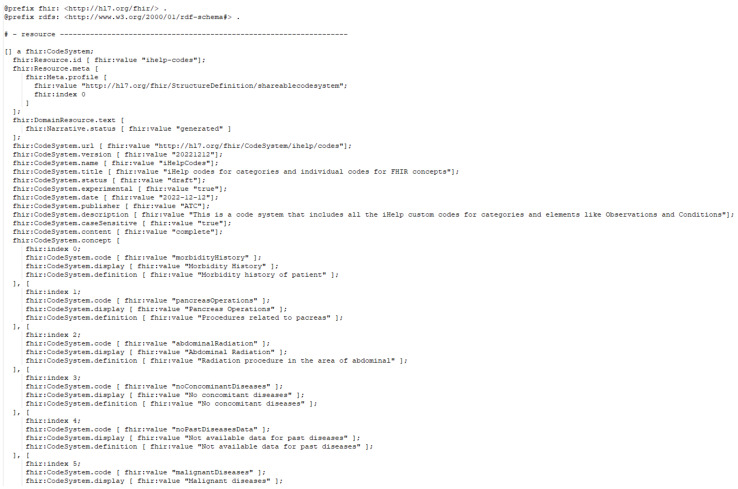
iHelp coding system sample.

Additionally, an instance of the iHelp CodeSystem, represented in TTL, is depicted in Figure 6, showcasing some of the custom iHelp codes, e.g., Morbidity History, Malignant Diseases, etc., used in the context of the HDM use case. The CodeSystem is defined based on the FHIR guidelines, so it is comprised of all the necessary definitions like version, title, description, etc., and, most importantly, the concepts that constitute the actual codes of the system.

### 2.5. Data Standardization and Qualification Methodology

The deployment of advanced healthcare analytical tools and frameworks not only results in the increased productivity of the healthcare professionals but also overall improved patient management and care. However, the analysis of data is mostly reliant on standardization and qualification of underlying data [40]. To this end, the proposed pipeline in the iHelp platform addresses these aspects by exploiting three (3) processing phases: the cleaning, the qualification, and the harmonization of the data. These phases are realized through the design and implementation of three (3) integrated subcomponents, i.e., the Data Cleaner, the Data Qualifier, and the Data Harmonizer, respectively, as depicted in Figure 2 and initially introduced in [41].

**Figure 7:**
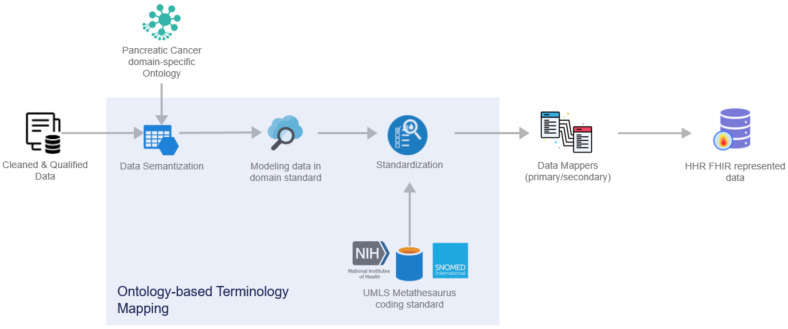
Data Harmonizer internal subcomponents.

In deeper detail, as soon as all the needed data are ingested into this pipeline by the Data Collection and Ingestion building block, the first two phases of this pipeline are responsible for the cleaning and quality assurance of the collected data. Thus, from the very beginning of the overall processing pipeline, it aims to clean all the collected data and to measure and evaluate the quality of both the connected data sources and their produced data through the exploitation of ML-based data-processing techniques. To successfully achieve that, the optimized pipeline exploits two (2) separate modules, the Data Cleaner subcomponent and the Data Qualifier subcomponent. Sequentially, in the harmonization phase, the interpretation and transformation of the collected, cleaned, and reliable data take place through the implementation and utilization of the Data Harmonizer. This component incorporates two (2) subcomponents, the Ontology-Based Terminology Mapping service and the Data Mappers, to further transform the cleaned and reliable data and to provide interoperable, harmonized, and transformed HL7 FHIR standard data, as depicted in Figure 7.

More specifically, the overall workflow of the Data Harmonizer can be encapsulated through the below steps:

- Cleaned and qualified data are semantically analyzed and mapped to concepts and instances of a domain-specific ontology that has been provided in the context of the iHelp project [32];
- Data are standardized into the project’s common data model and domain standard;
- The PyMedTermino [42] and UMLS metathesaurus [43] are utilized, offering a wide collection of terminology services. The different terminologies, coding standards, and vocabularies that are offered through these systems are utilized to further transform the medical terms between terminologies in a controlled and supervised manner;
- Finally, standardized and harmonized data are fed into the Primary and Secondary Data Mappers to be transformed into the HHR FHIR-compliant format. The actual realization of the conceptual HHR model is performed with the assistance of the FHIR mappers. The implementation of them is based on the Java library of HAPI FHIR and exposes APIs that the Data Harmonizer component can consume [44].

To this end, the proposed pipeline facilitates the standardization and qualification of the heterogeneous primary and secondary data coming from multiple health-related sources and provides data in a unique and globally recognized standard and format such as the HL7 FHIR. At this point, it should be noted that the HL7 FHIR standard employs a structured approach for representing healthcare information, particularly in the context of numerical and categorical data in the primary data. In that context, for quantitative data, such as clinical measurements or laboratory values, the valueQuantity field within the Observation resource encapsulates both the numeric parameter and its corresponding units, adhering to a standardized system. On the contrary, categorical information, including diagnoses and patient conditions, is encoded using the valueCodeableConcept field, ensuring semantic interoperability by referencing standardized coding systems like SNOMED, which is used in the scope of this research work. This standardization approach can be also applied to the secondary data that are collected and processed and particularly to the questionnaire responses. For capturing this type of patient-reported information, HL7 FHIR includes the Questionnaire and QuestionnaireResponse resources, which can be used to handle both numerical and categorical responses by using the answer field. This systematic and standardized representation allows for a robust and consistent exchange of both quantitative and qualitative healthcare data, promoting interoperability across diverse healthcare systems. 

## 3. Results

In this section, the performance of the core components of the proposed mechanism is analyzed together with its potential for introducing integrated and standardized data in HHR format. In deep detail, this article focuses on evaluating the effectiveness of the operation of the different subcomponents integrated in the Data Ingestion Pipeline of the iHelp platform. It should be noted that the evaluated components have been developed in Java SE and Python programming languages, showcasing the generalization and improved integration of the introduced mechanism with widely used frameworks.

### 3.1. Use Case Description

To evaluate the proposed mechanism, both primary and secondary data from the HDM pilot have been utilized. The HDM use case is focused on predicting the risk of pancreatic cancer, while secondary data are gathered through the utilization of the Healthentia platform to analyze the impact of changes in lifestyle and habits on the identified risk factors. At its initial stage, this pilot obtained patients’ medical records from the hospital’s local Electronical Health Records (EHRs). The data extraction was performed in CSV files and following the Observational Medical Outcomes Partnership (OMOP) Common Data Model (CDM) [45]. It should be noted that these data represent patients that are separated into two main groups:

- Individuals that are directly involved in the iHelp project for further monitoring and follow-up by the HCPs of the HDM. Out of these individuals:
  ◦ Six are patients already diagnosed with pancreatic cancer. In the context of this pilot study, they provided their medical records and one single blood sample for the performance of epigenomic analytics;
  ◦ Thirteen are patients without pancreatic cancer. In addition to their medical records, a blood sample was provided every 3 months, and lifestyle data were collected through a 9-month monitoring phase based on the wearable devices and periodic questionnaires through the Healthentia platform.
- Individuals not directly inside the program:
  ◦ An extraction of medical records from around 90 thousand patients is anonymized and provided to the iHelp platform.

**Figure 8:**
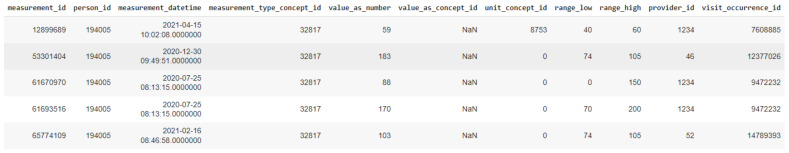
A sample of the Measurements dataset.

It should be noted that, in the HDM pilot, no bias has been identified in the examined data, and the 90 thousand patients represent the full population of the geographic area that is assigned to the hospital. The data that are ingested in the iHelp platform are fully anonymized, and the study is performed under the approval of the hospital’s Ethical Committee. Following the OMOP standard, a collection of seven (7) different primary datasets is produced, provided, and examined in the context of this pilot study based on the respective information, as presented in Table 1. A sample from one these primary datasets related to the different measurements is also depicted in Figure 8.

### 3.2. Data Collection and Ingestion

The Data Collection and Ingestion mechanism in the iHelp platform encompasses all tasks associated with collecting, validating, and ingesting both primary and secondary data into the iHelp platform. The primary data are directly captured and ingested by the Data Capture Gateway through the implementation and utilization of different data connectors. Afterwards, the initial validation of the integrity of the data is achieved through the utilization of the Avro Schema, which also requires the use of an Avro Schema Registry, which allows only the transmission of the number of bytes that concerns the data themselves, thus minimizing the overall size of the data elements, as well as the time needed for their ingestion and overall processing [46]. For instance, the time that is needed for the whole Measurements dataset from its initial capture to its final transformation as HHR standardized data and storage in the platform’s data storage is 5 min and 26 s. The schema of the dataset is transformed by the Data Capture Gateway in an Avro Schema compatible format, which boosts the interoperability and has a well-known standard to be further used by other functions involved in the data ingestion and processing process. 

**Figure 9:**
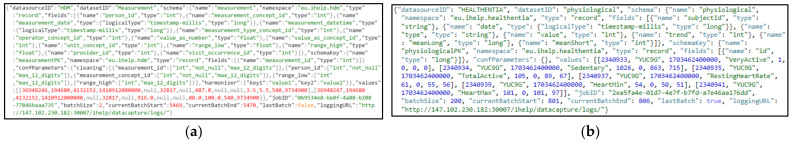
Messages interexchanged between the components of the Data Ingestion Pipeline: (**a**) message including primary data; (**b**) message including secondary data.

Moreover, the intermediate software components that formulate the Data Ingestion Pipeline are domain and schema agnostic. This means that a flexible ingestion pipeline is established as each function can consume and produce data from corresponding Kafka topics in a dynamic manner and without any prior knowledge of the data. The respective information is passed to each subcomponent through these messages, enabling all subcomponents to communicate using this common data format. This format is designed to be highly interpretable and in such a way in order to be irrespective of the dataset, schema, and type of data that are contained in these messages. An example of such messages is depicted in Figure 9, which shows a message with primary data derived from the Measurements dataset with a batch of two elements, as well as a message containing secondary data relating to physiological measurements as derived from the Healthentia platform. 

The most important attributes of these JSON objects and messages are presented below:

- datasourceID: the name of the data provider;
- datasetID: the name of the dataset;
- schema: the schema of the value of the tuples, defined in Avro Schema;
- schemaKey: the schema of the key of the tuples, defined in Avro Schema;
- batchSize: the batch size;
- currentBatchStart: the index of the first element of the batch in the overall dataset;
- currentBatchEnd: the index of the last element of the batch in the overall dataset;
- confParameters: the configuration parameters required by each of the intermediate functions. It includes an array of data parameters packed in JSON format, where each JSON can be interpreted by the corresponding function. These parameters are being passed to each of the intermediate functions, and each one of those can retrieve the ones of their interest. For instance, specific cleaning rules have been set by the data provider concerning specific data attributes, as depicted in Figure 9a. These rules are consumed by the Data Cleaner to perform the necessary cleaning and validation actions on the data;
- values: a list of the exploitable data and their different values per each record.

These messages are exchanged between different subcomponents of the Data Ingestion Pipeline by utilizing the Kafka message broker, as analyzed before. 

**Figure 10:**
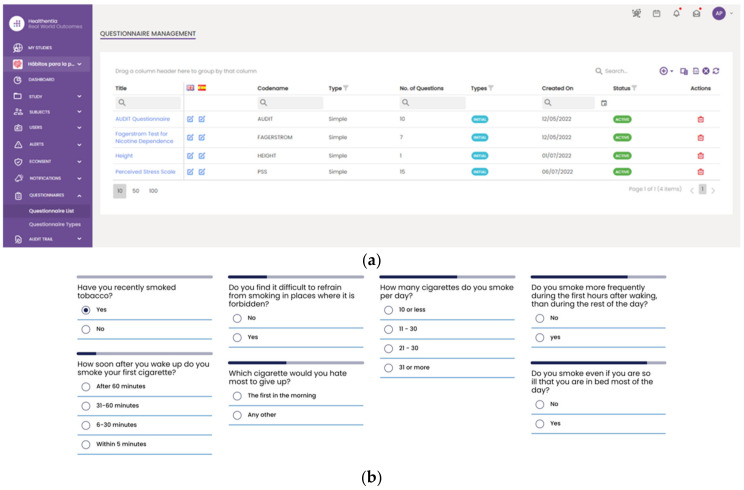
Using questionnaires in the HDM study. (**a**) List of defined questionnaires on the Healthentia portal; and (**b**) a patient answering the Fagerstrom questionnaire in the mobile app.

However, a slightly different procedure is followed for the collection phase of the secondary data. These data are initially collected using the Healthentia mobile application [37] rather than directly fetched by the Data Capture Gateway. It is important to mention that the Healthentia mobile application gives access to answers to different questionnaires that are used for self-assessment, while activity trackers collect individuals’ physiological and exercise data. The data collected and processed in the context of this paper are related to a six (6)-month period, i.e., from 1 June 2022 to 31 December 2022, monitoring the daily activities of the individuals participating in the study. The questionnaires are selected by HCPs and are defined in the Healthentia portal, together with the timing used for pushing them to patients automatically. More specifically, Figure 10 depicts the questionnaires defined for the HDM study, as well as how such a questionnaire is answered by a patient in the Healthentia mobile app.

**Figure 11:**
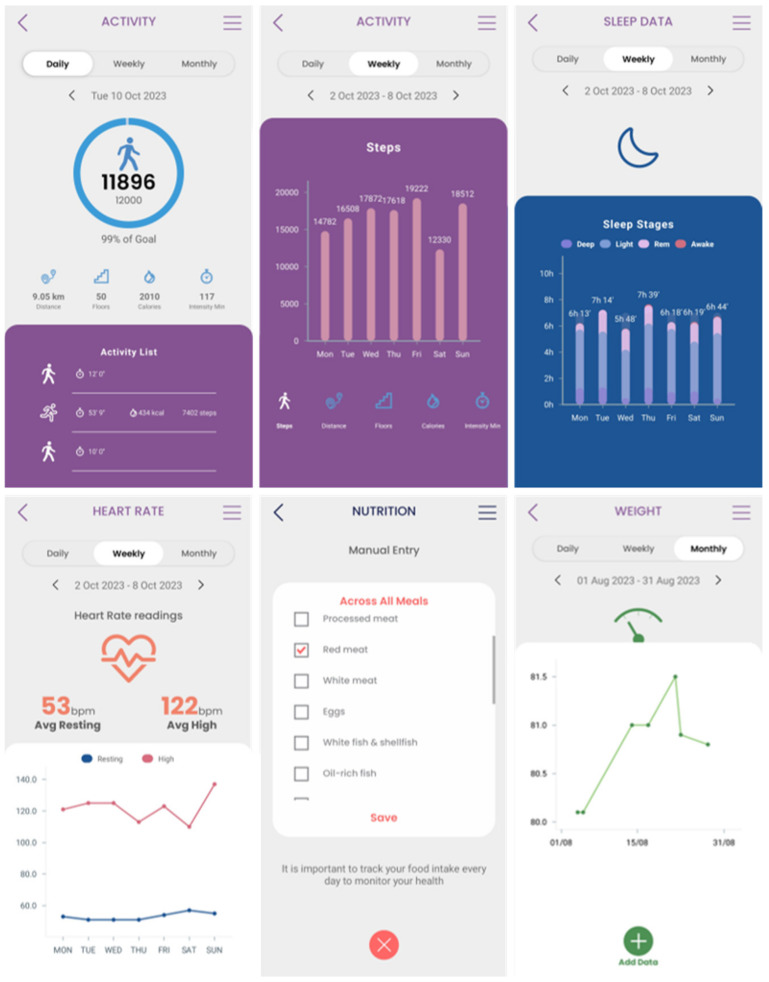
Entering and visualizing data in the Healthentia mobile app.

The different widgets accessible from the main dashboard of the mobile app (see Figure 11) give access to data entry functionalities and visualizations of data collected from activity trackers (physical activity, sleep, and heart info), other devices (like scales), and the nutrition widget, as shown in Figure 11.

Regarding the information related to the answers, exercises, and physiological data, a specific connector has been implemented in the Data Capture Gateway. Depending on the type of dataset, it connects to the corresponding REST API provided by Healthentia and receives the respective list of information. 

### 3.3. Data Standardization and Qualification towards Holistic Health Records

This sub-mechanism is evaluated on real-world primary and secondary data which have been provided in the context of the iHelp project [32], where clinical data of pancreatic cancer patients are analyzed to provide personalized recommendations.

At first, the Data Cleaner component is utilized as an integrated component of the Data Standardization and Qualification mechanism, and its main objective is to deliver the software implementation that provides the assurance that the provided data coming from several heterogeneous data sources are clean and complete, to the extent possible. This component is designed to minimize and filter the non-important data, thus improving the data quality and importance by implementing ML techniques, such as data imputation and outlier detection and deletion. Hence, different data manipulation and cleaning techniques were performed to handle missing values and any other inconsistencies in the examined datasets. In deeper detail, the imputation step was implemented by using the K-Nearest Neighbor (KNN) algorithm to fill in the missing values in the respective columns, taking into consideration the different groups of patients based on their age and sex type for improved performance and appropriate imputation of missing values considering demographic-specific patterns [47]. Handling the mixed data types was another essential step in the data-processing pipeline for identifying and rectifying columns with mixed data types (e.g., numerical and string values), ensuring data uniformity. In addition, the outlier detection and removal phases were implemented through the utilization of two different techniques to effectively handle all different values. More specifically, univariate outlier detection using the z-score threshold [48] and the Density-Based Spatial Clustering of Applications with Noise (DBSCAN) [49] algorithm were utilized and evaluated in relation to the secondary and primary data, respectively, to identify and handle anomalous values that could skew analysis results. The reason behind this approach is that DBSCAN is more applicable to cluster analysis data applications than to anomaly detection. The latter is preferred for the secondary data that were collected through the wearables to identify anomalies in the measurements provided by these sensors, while the DBSCAN algorithm is applied to the primary data to leverage the information that can be derived from the analysis of patient-specific groups based on their demographic characteristics. To address a portion of these challenges, referring mainly to reducing the complexity and facilitating the analysis of large datasets, the applied ML-based data-cleaning procedures attempt to improve the data quality and to enhance the analytical outcomes since wrong data can drive an organization to wrong decisions and poor conclusions. To this end, this component seeks to assure the incoming data’s accuracy, integrity, and quality. The results of its application are presented in the below table, i.e., Table 2.

According to the Data Cleaner results, only a few dataset records were eventually dropped since the initial datasets provided by the EHR system of the hospital had good consistency overall and a low number of empty or erroneous values, especially in features with high importance in the final result and analysis. The same applies in the collection of physiological and questionnaire secondary data from the wearables and the mobile application. Small disparities were observed in the physiological and exercise data that were collected through the wearables. These erroneous observations relating to the data were related to false measurements received with regard to steps, sleep, and other lifestyle habits. It is also worth mentioning that the questionnaires are based on multiple answers and Likert scale answers; thus, no errors were observed. As depicted in the above table, our ML-based data-cleaning techniques successfully achieved the correction of erroneous records, resulting in more reliable and qualified data that further enhanced the capabilities and accuracies of the analytical models. Generally speaking, the purpose of a health policy is to provide standardization in daily operational activities. Given that a health policy is intended to establish the basis for the delivery of safe and cost-effective quality care, only the most understandable and clearly set data instances should be provided.

Afterwards, the Data Qualifier component classifies data sources as reliable or non-reliable both during the primary and secondary data injection. A data source is classified as reliable when the datasets received from this source are considered correct; otherwise, it is considered as non-reliable. To test this feature, this component acquires both the cleaned and faulty data produced by the Data Cleaner component. The results from the utilization of this subcomponent in the HDM use case are presented in Table 3.

The Data Qualifier subcomponent is divided into the two different sub-functions shown. The Dataset Qualifier sub-function processes the cleaned dataset and the faulty data to evaluate the dataset reliability. For that purpose, it calculates the size of the dataset and takes into account the number of cleaned data in that dataset. The reliability is provided for the whole dataset. These values range from 0 to 1, where 1 is the highest reliability and 0 the lowest. First, it calculates the reliability of each column; per column, the reliability is one minus the total number of faulty values divided by the total number of occurrences of the column in the dataset. Afterwards, the Datasource Qualifier sub-function calculates the reliability of the specific data source that produces the data. For instance, a wearable device monitors the heartbeats, sleeping time, number of steps, and blood pressure, among other metrics. If the heartbeat values are considered faulty for a batch of data or period of time, the heartbeat sensor is considered not reliable. As depicted in the above table, all the processed datasets are of high quality, thus highlighting the overall acceptance and reliability of the data sources and ensuring improved decision making and performance of the analytical results.

**Figure 12:**
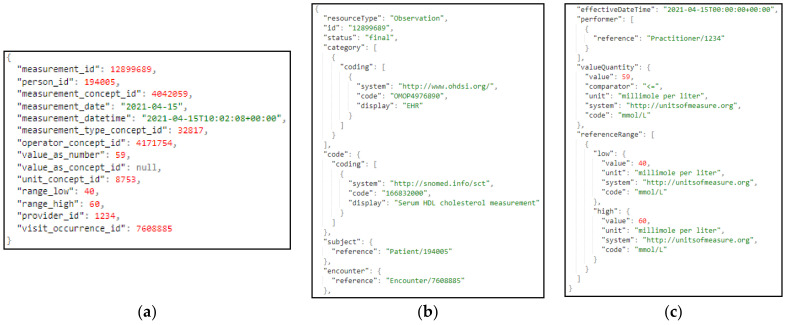
Sample transformation of raw primary data to the HHR format via the iHelp mappers, where (**a**) represents the raw primary data as they are collected by the hospital; (**b**,**c**) depict the data transformed to HHR format.

With regard to the Data Harmonizer component, initially, it translates the hospital data coming in into SNOMED concepts, and these concepts are fed to the mappers for further analysis. Coupled with the utilization of the FHIR ontology, the Data Harmonizer component provides a set of intelligent services to manage terminology resources and make the data semantically interoperable. In addition, it provides a set of operations for widely used and known medical terminologies used for the coding of medical knowledge, such as LOINC [50], ICD-10 [51], and SNOMED, which further enhance the information structures that are provided as outputs from the Data Harmonizer component. In addition, it provides the flexibility to the whole iHelp platform to utilize new releases of terminologies and to provide mappings or translations between different terminologies and standards. The latter is addressed through the extensible searching and querying functionality for specific elements of the well-established terminologies and standards. The mappers receive as input the harmonized and semantic interoperable data and then transform these concepts into the appropriate FHIR elements, grouping the elements as needed. Finally, an FHIR Bundle containing the mapped data is sent back to the Data Harmonizer for fusion of the HHR-based modeled data to the platform’s data storage. A sample harmonization of raw primary data to HHR data is depicted in Figure 12.

**Figure 13:**
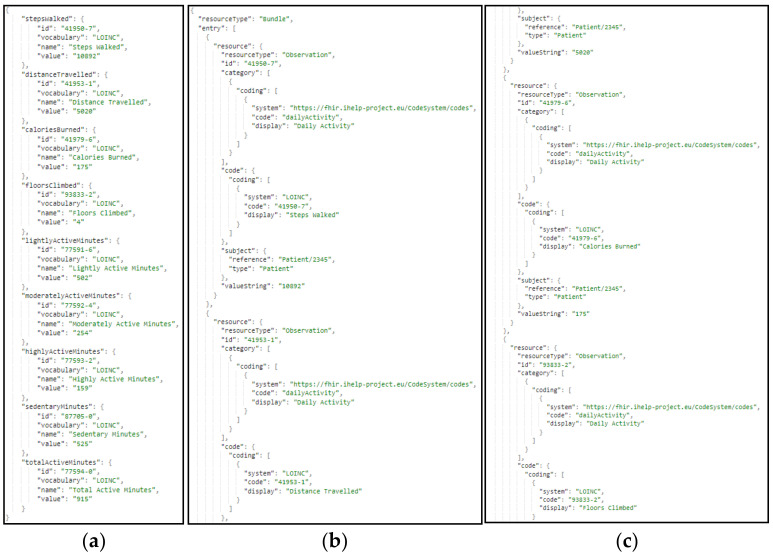
Sample transformation of secondary data to HHR format, via the HHR secondary data mapper, where: (**a**) represents the raw secondary data as they are collected by the wearable device; (**b**,**c**) depict the data transformed to HHR format.

The same approach and transformation are followed in the case of the ingestion of secondary data. These data represent lifestyle and behavioral aspects of the patients’ life. These data are gathered through wearable devices, as well as answers to questionnaires and nutrition-related information. In Figure 13, a sample harmonization of raw secondary data to the standardized HHR model is depicted. More specifically, in this figure (Figure 13), a sample transformation of raw secondary data to the HHR format via the iHelp mappers is represented, where (a) represents the raw secondary data related to the daily activity and as they are collected by the individual wearable device; and (b,c) depict the daily activity data of the individual transformed to HHR format mapped to an Observation resource type. In that context, the Data Harmonizer subcomponent implements all the processes that utilize widely used and known coding standards and terminologies coupled with domain-specific ontologies. It also further facilitates the aggregation of the distributed heterogeneous, cleaned, and qualified data and provides the final harmonized data mapped into the globally recognized FHIR standard and the common HHR format.

## 4. Discussion

The overall iHelp platform contributes to the shift from acute-based to evidence-based care by providing improved access to patient-related information. Through the integration of innovative data management, ML, and Semantic Web techniques, HCPs can have access to advanced knowledge related to each patient they are treating. In particular, the utilization of patients’ integrated data, in the form of HHRs, is facilitated through the proposed mechanism and acts as a crucial preliminary step towards the provision of improved clinical knowledge, integrated information about the patient’s status, and non-fragmented and interoperable healthcare data. The latter can result in an improved understanding of underlying causal risk factors for pancreatic cancer as these integrated, qualified, and standardized data can be leveraged in later stages from advanced AI models to provide decision support in the form of early risk predictions as well as personalized prevention and intervention measures. Consequently, this can lead to improved identification and understanding of the key risk factors contributing to the development of pancreatic cancer, which are typically difficult to study only through primary data. Among their main advantages, the integrated subcomponents of the iHelp Data Ingestion mechanism allow HCPs to synchronously monitor the progress of their patients and achieve better coordination of their care responsibilities through the provision of integrated and HHR-transformed data. To this end, this mechanism gives the HCPs a more effective approach, allowing them to administer care through better planning, to better manage decisions and mitigation plans through the continuous and substantive flow of integrated health-related data, to better prepare for providing treatment and recommendations, and to better manage the integrated and harmonized health data in the HHR format. Based on the availability of HHRs, the analysis and identification of the causal risk factors become easier and more effective, contributing to increased understanding of pancreatic-cancer-related risks, improved early diagnosis, and the provision of enhanced personalized prevention and mitigation plans. 

Among its indirect impacts, by effectively gathering data both from individuals’ EHRs and personal IoMT devices, collective community knowledge could be extracted, achieving a significant dual goal: (i) fusing, collecting, and analyzing information from multiple sources to generate valuable knowledge and actionable insights for the HCPs, and (ii) facilitating the development of personalized and efficient prevention plans and decisions [52]. The impact of such solutions using community knowledge, which is collective, in the domain of healthcare is apparent since information sharing has changed its overall approach towards better diagnostics and improved QoL [53].

It is worth mentioning that the overall iHelp platform has been designed and implemented in such a way that it allows several cases of extensibility. The platform’s validation and evaluation are performed in five (5) different use cases and scenarios in the context of the iHelp project. At first, it allows for extensibility in terms of new datasets since the functionalities of the Data Collection and Integration and Standardization and Qualification building blocks, presented in this research work, enable new datasets to be directly ingested into the internal datastore by following a standard path, finally being represented in the platform as HHRs. Apart from this, the platform allows for extensibility in terms of new data sources, as demonstrated through the integration of the Healthentia mobile application and wearable devices as new data sources, from which data can be gathered and utilized for decision making. As soon as these new data sources are identified, the overall data ingestion flow can be followed, as described in the abovementioned extensibility case.

In this paper, only a specific use case and data from one hospital were examined, verifying the functionalities of the platform, which could be considered a potential limitation. Targeting this, and concerning future research and further updates on the introduced mechanism, it is among future plans to evaluate the platform with more use case scenarios and different types of data, e.g., from other cancer types. Furthermore, we aim to disseminate the outcomes of the iHelp project to receive valuable feedback on the platform and its usage scenarios and to adapt the implemented components to the different needs of the healthcare stakeholders. The latter will facilitate the development of a holistic and multidisciplinary Health Technology Assessment (HTA) approach considering multiple parameters and standardized metrics and KPIs. It will combine outcomes of Clinical Studies and Randomized Control Trials (RCTs) with Real-World Evidence (RWE) from the different use cases and scenarios, on which the platform will be utilized and evaluated in the context of the iHelp project [32].

## 5. Conclusions

In the realm of healthcare, today’s HCPs are presented with remarkable opportunities to gather and manage comprehensive digital health records, drawing from various sources, including records of individuals’ lifestyle behaviors and habits, EHRs, and medical data repositories. This variety of data has the potential to facilitate a shift towards data-driven healthcare practices and AI-driven healthcare analytics and decisions. The integration of AI in the healthcare decision-making (e.g., monitoring, real-time decision support) phase is still evolving, with persistent challenges related to the interoperability of the data and the trustworthiness and explainability of the models when aiming to develop improved and more interpretable prevention and intervention strategies.

This paper has discussed these challenges and presented solutions that can advance the state of data-driven personalized decision support systems. The main contribution of the paper is the introduction of an advanced mechanism for health-related data processing integrating Semantic Web and ML techniques, also leveraging the potential derived from the utilization of integrated primary and secondary data in the HHR format. The viability of this approach has been evaluated through heterogeneous healthcare datasets pertaining to risk identification and individual monitoring and care planning.

In this paper, the applicability of the introduced mechanism was validated on a specific use case and with data derived from a single hospital’s EHR system (primary) and one type of wearable (Garmin devices), which could be considered a potential limitation. Hence, its further evaluation with data collected from different data providers is among our future research plans and further updates on the mechanism. The latter will be also performed in the context of the iHelp project, where the overall platform’s validation and evaluation are performed in five (5) different use cases and scenarios related to pancreatic cancer. It should be noted that this is a work in progress, and more results and improvements will be published in the future after testing it with more healthcare systems and wearable devices to verify its global applicability. In addition, taking into consideration the ethical aspects that can be raised within the whole data lifecycle, we focus on the integration of this mechanism with an advanced data-logging mechanism and explainable AI (XAI) techniques, such as the LIME framework [54] and decision trees [55], to provide clear and interpretable insights into the data-cleaning, qualification, and standardization processes that are applied in our proposed end-to-end data-processing pipeline. Finally, with regard to the introduced ontology, it is among our future plans to refine, expand, and dynamically update it. Our intention is to finalize and publish the ontology, making it available for further exploitation by the research and healthcare communities and aiming to foster cross-disciplinary collaboration between experts. In that direction, we also are pursuing the adoption of the HHR format and its corresponding suggested coding system as an official FHIR Extension. Through these steps, our approach and mechanism will offer a powerful tool for the development of patient-centric, effective, and sustainable solutions in the healthcare domain.

## Figures and Tables

**Table 1:** HDM dataset descriptions.

Dataset Name	No. of Records	No. of Attributes	Dataset Size (in MB)	Dataset Type
Measurements	3,252,920	17	564.1	CSV
Observations	339,925	14	55.4	CSV
Person	99,019	8	2.8	CSV
Drug Exposure	5,411,914	13	779.9	CSV
Condition	1,833,512	14	248.5	CSV
Visit Occurrence	5,205,819	13	727.5	CSV
Procedure	602,351	12	147.5	CSV

**Table 2:** Overall cleaning results.

Dataset Name	Initial Records	Erroneous Records	Corrected Records	Deleted Records	Faulty Data (%)
Measurements	3,252,920	1049	1011	38	0.0011%
Observations	339,925	139	122	17	0.005%
Person	99,019	144	119	25	0.025%
Drug Exposure	5,411,914	635	598	37	0.00068%
Condition	1,833,512	336	325	11	0.0006%
Visit Occurrence	5,205,819	208	169	39	0.0007%
Procedure	602,351	180	162	18	0.0029%
Exercises Secondary Data	5344	3	3	0	0.00%
Physiological Secondary Data	22,136	9	6	3	0.0135%
Questionnaire Secondary Data	2721	0	0	0	0.00%

**Table 3:** Data Qualifier outcomes.

Dataset Name	Data Source	Dataset Reliability Score	Datasource Reliability Score
Measurements	HDM Hospital	98%	Reliable
Observations	HDM Hospital	97%	Reliable
Person	HDM Hospital	95%	Reliable
Drug Exposure	HDM Hospital	98%	Reliable
Condition	HDM Hospital	98%	Reliable
Visit Occurrence	HDM Hospital	97%	Reliable
Procedure	HDM Hospital	96%	Reliable
Secondary Data (derived from Healthentia)	Healthentia Platform	99%	Reliable

## Data Availability

Data are contained within the article.
